# Hepatitis B virus infection on Kwajalein Atoll, Marshall Islands: a seroprevalence, knowledge and attitudes study

**DOI:** 10.5365/wpsar.2024.15.1.1042

**Published:** 2024-02-21

**Authors:** Melaia Lawanivalu, Anaseini Ratu, Glorine A Jeadrik, Masoud Mohammadnezhad, Aneley Getahun Strobel

**Affiliations:** a Ebeye Public Health Department, Leroij Kitlang Memorial Health Center – Ministry of Health and Human Services, Majuro, Marshall Islands.; b School of Public Health and Primary Care, College of Medicine, Nursing and Health Sciences, Fiji National University, Suva, Fiji.; c Kwajalein Atoll Health Care Bureau, Leroij Kitlang Memorial Health Center – Ministry of Health and Human Services, Majuro, Marshall Islands.; d School of Nursing and Healthcare Leadership, University of Bradford, Bradford, United Kingdom of Great Britain and Northern Ireland.; e Department of Microbiology and Immunology, The University of Melbourne at the Peter Doherty Institute for Infection and Immunity, Melbourne, Victoria, Australia.

## Abstract

**Objective:**

A study was conducted to determine the seroprevalence of chronic hepatitis B virus (HBV) infection among children and their mothers on Kwajalein Atoll in the Marshall Islands two decades after routine vaccination was introduced in the 1990s. Mothers’ knowledge and attitudes towards HBV disease and vaccination were also assessed.

**Methods:**

Results of a national seroprevalence survey conducted in 2016–2017 and antenatal records were used to determine the prevalence of HBV seropositivity in children aged 6–8 years and their biological mothers. The associations between demographic, social and vaccination-related factors and seropositivity were explored using Fisher’s exact tests.

**Results:**

HBV seroprevalence was 0.3% in children and 6.8% in their mothers (during pregnancy). Coverage of timely HBV vaccination was 90.3% for the birth dose and was significantly associated with factors related to place of residence (*P* < 0.001), place of birth (*P* < 0.001) and number of antenatal visits (*P* < 0.001). Maternal attitudes towards infant vaccination and antenatal screening were largely positive (95.8% and 96.7%, respectively) despite low vaccination rates (20.9%) among mothers. Knowledge levels were low for disease complications, treatment and transmission.

**Discussion:**

Prevalence of HBV in children and mothers residing on Kwajalein Atoll in 2016–2017 was lower than the national average for the Marshall Islands. Timely birth dose administration appears to have been effective in preventing mother-to-child transmission of HBV in this setting and should be promoted in remote settings where antiviral therapy is not available. Provision of out-of-cold-chain HBV vaccines should be considered to improve access in remote settings.

Prior to the introduction of the hepatitis B virus (HBV) vaccine in the 1980s, chronic HBV infection was highly endemic among countries in the World Health Organization (WHO) Western Pacific Region, with prevalence typically in excess of 8%. (*1*-*3*) The Region’s goals for HBV disease control are < 0.1% prevalence in 5-year-olds by 2030 and > 95% vaccination coverage for birth and third doses. (*4*, *5*)

Many Pacific island countries and areas (PICs) have reported significant reductions in HBV prevalence following the introduction of routine HBV vaccination. (*6*-*8*) However, timely, reliable and disaggregated estimates of disease prevalence are lacking for many Member States in the Region. (*9*, *10*) Reported estimates for HBV infection rates tend to be based on infrequent seroprevalence surveys (*10*, *11*) and are often expressed in terms of national averages that mask the within-country variations that likely exist across island groups in many PICs.

Existing strategies for HBV disease control in the Region emphasize the need for more granular, contextual epidemiological estimates of disease burdens in Member States and the need to expand disease control efforts beyond immunization once coverage targets have been achieved. Significant challenges exist in the delivery of both vaccines and treatment for HBV in PICs, including lack of resources, geographical dispersion, limited infrastructure and sociocultural barriers to immunization. (*1*, *9*, *12*, *13*)

The self-governing Marshall Islands comprise 34 low-lying atolls and islands, (*14*) with a population of 42 418. (*15*) The majority of the population resides in two urban centres on large atolls, 430 km apart: Majuro on Majuro Atoll (55%) and Ebeye on Kwajalein Atoll (23%). (*15*) A United States military base is present on Kwajalein Atoll but remains largely segregated from the Marshallese population.

Data on the seroprevalence of chronic HBV infection in the Marshall Islands are limited. The most recent available data are from a national seroprevalence survey conducted during the 2016–2017 school year. (*1*) According to this survey, the prevalence of HBV among first graders (children aged 6–8 years) was 1.2%. (*1*) Comparisons with earlier seroprevalence surveys, and thus analyses of temporal trends in hepatitis B disease burden, are problematic as these were conducted in different study populations. Available data from the 1980s showed a HBV prevalence in excess of 8% among adult blood donors, indicating previously high endemicity. (*2*, *16*) According to data from 2007, seropositivity among children in first grade and unrelated prenatal women from the two main urban centres (i.e. Majuro and Ebeye combined) averaged 1.8% and 9.5%, respectively. (*17*) While the 2016–2017 national seroprevalence survey included children from Kwajalein Atoll, reported results were not disaggregated by geographic areas. Currently, there is no systematic screening for HBV in adults in the Marshall Islands and no current estimates of seroprevalence in prenatal women (*17*) or HBV vaccine coverage among adults. (*2*, *16*)

Timely HBV birth dose vaccine coverage for the whole of the Marshall Islands in 2016 was 87%; third dose vaccine coverage was 76%. (*18*) However, immunization rates are known to differ between atolls, with the outer remote islands typically having lower coverage rates. (*9*, *17*) This may be due to a higher number of non-hospital births in the more remote islands, which often means the birth dose is delayed. (*9*, *17*) While information about HBV disease awareness and attitudes towards vaccination is generally lacking among the adult population in the Marshall Islands, (*19*) one study conducted in another PIC suggests a baseline knowledge of HBV in up to 60% of adults. (*12*)

Nearly a quarter (23%) of the national population resides on Kwajalein Atoll, the largest and most densely populated atoll in the Marshall Islands. However, the atoll is relatively remote from the nation’s capital, Majuro, and has limited infrastructure and resources. (*15*) The risk of HBV disease transmission is high on Kwajalein Atoll due to its high population density, (*15*) low HBV vaccine coverage among adults, (*13*) and high prevalence of high-risk behaviours for transmission in resident adolescents and young adults. (*13*, *20*) Evaluation of disease prevalence among children and mothers on the atoll is needed to improve understanding of the hepatitis B disease burden and to inform strategies for expanding disease control measures. Assessment of knowledge and attitudes is also needed to support implementation of appropriate measures for improving vaccine uptake and screening. (*19*, *21*)

The objective of this study was therefore to determine the seroprevalence of chronic HBV infection among young children and their mothers on Kwajalein Atoll in the Marshall Islands two decades post vaccine introduction and to assess the knowledge and attitudes of mothers towards HBV disease and vaccination.

## Methods

### Study setting

Kwajalein Atoll comprises 90 islets and has a population of 9739, half of which resides in Ebeye, the one major urban centre located on the main islet. (*15*) The population is predominantly native Marshallese. (*15*) The atoll has a 55-bed hospital, three primary care facilities (dispensaries) and 11 primary schools. Across the Marshall Islands, prenatal care is accessed by 81% of pregnant women. (*22*) At 95%, primary school enrolment on Kwajalein Atoll is higher than the national average of 85% (2016 data). (*23*)

On average, Kwajalein Atoll has 255 live births per year, the majority of which (95%) are delivered in Ebeye Hospital. HBV vaccination services are provided by immunization programme staff of the Ebeye Public Health Department. Routine childhood vaccination for HBV was introduced in Kwajalein Atoll in 1992 and administration of a timely birth dose (i.e. within 24 hours of birth) began in 1998. (*17*) Reported HBV vaccination coverage for the 2010 birth cohort was 99% for a timely birth dose and 97% for a complete course of three doses. (*10*) At the time of this study, immunoglobulin therapy for treatment of neonates delivered from seropositive mothers was not available in the Marshall Islands.

### Study participants

First grade students aged between 6 and 8 years and their biological mothers were selected for this study. All first grade students enrolled in all 11 primary schools on Kwajalein Atoll in November 2016 were recruited to this study, apart from one who migrated and was therefore excluded. The choice to conduct the study in first grade students, who are older than the target age group recommended by the WHO for seroprevalence surveys (5 years), was a pragmatic one, given that school-aged children are easier to reach through school-based immunization activities.

The mothers of the first grade students were identified through school records. As there were two sets of twins in the study cohort of first grade students, the number of biological mothers was slightly lower (*n* = 296). An additional 64 mothers were randomly selected from mothers of second grade students attending the two largest elementary schools on Kwajalein Atoll to increase the number of knowledge and attitude survey participants to meet the required sample size (*n* = 357, assuming 80% power and an expected prevalence of baseline knowledge of 60%). (*21*)

### Data collection

For all first graders, seroprevalence data were obtained from the results of the national survey conducted in 2016–2017. Demographic data (age, sex, ethnicity, place of birth, parents’ names) were also extracted from the survey data and cross-checked against their birth and vaccination records for verification purposes.

Demographic data and perinatal HBsAg screening results for biological mothers were extracted from available antenatal, medical and immunization records. Structured face-to-face interviews were conducted from July to August 2018 with the mothers to assess their knowledge and attitudes towards HBV infection and vaccination. The interviews were conducted by public health nurses using a standardized data collection tool at participants’ residences. Demographic information on mothers was also collected during the interviews to verify data in their antenatal records.

### Data analysis

Children were considered seropositive for HBV infection if according to the results of the national seroprevalence survey they had tested positive for HBsAg. The national survey used a rapid test kit (Abbott Determine; Chiba, Japan) to test for HBsAg from whole blood from finger pricks; all positive results were verified at a medical laboratory in Hawaii. A timely HBV birth dose vaccination was defined as receipt of HBV vaccine within 24 hours of birth as documented in birth records. Mothers who had a positive HBsAg test result recorded during their pregnancy were defined as having a chronic HBV infection. For mothers with no antenatal record (*n* = 60) for the pregnancy of interest, HBV status was assessed in subsequent pregnancies and other sociodemographic data were extracted from their medical records. Maternal chronic HBV infection was assessed in two age groups, above and below 25 years, to distinguish those born before and after HBV vaccine introduction to the Marshall Islands.

Characteristics of the study population were summarized in a descriptive analysis using counts, means, standard deviations and proportions, as appropriate. Associations between dependent (HBV infection and vaccination status) and independent variables (sociodemographic and clinical factors) were explored and Fisher’s exact tests used to determine the significance of these relationships. Statistical significance was set at *P* < 0.05.

The knowledge and attitude survey was structured around 10 knowledge questions and six attitude questions, which required “yes” or “no” answers for both sets of questions. The survey was designed to be as simple as possible to account for low education levels and the need to conduct interviews in the Marshallese language. Overall knowledge and attitude scores for each participant were calculated as the proportion of “yes” answers. A knowledge score of < 6 and attitude score of < 4 was considered poor. The proportion of mothers showing a positive response for specific knowledge and attitude items was calculated with 95% confidence intervals (CIs). All analyses were conducted using Stata version 17.

### Informed consent

The seroprevalence survey of the children was conducted as part of a national campaign, and informed consent was obtained from parents and guardians before their inclusion. Prior written informed consent was obtained from women who participated in the survey of knowledge and attitudes.

## Results

### Seroprevalence survey

Seroprevalence survey data for 2016–2017 were available for a total of 298 first grade students from Kwajalein Atoll. Of these, 57.0% (170/298) were males, the mean age was 6 years (range, 6–8 years; SD = 0.6) and most (87.6%) were residents of Ebeye. Nearly all first graders (90.6%) were born in hospital. A similar proportion (90.3%) received a timely HBV vaccine birth dose; 58.1% completed the third dose by 6 months of age (**Table 1**). Only one child was seropositive for HBsAg (0.3%, 95% CI: 0.32–0.99%) and had a seropositive mother. Further analysis for factors associated with seropositivity in children was not attempted.

**Table 1:** Characteristics of first grade children from Kwajalein Atoll included in the 2016–2017 national seroprevalence study of HBV infection, by infection status

Characteristic	All children (*n* = 298) *n*(%)	Seropositive (*n* = 1) *n*(%)	Seronegative (*n* = 297) *n*(%)
**Age (years)**			
6	177 (59.4)	1 (100)	176 (59.3)
7	101 (33.9)	0 (0)	101 (34.0)
8	20 (6.7)	0 (0)	20 (6.7)
**Sex**			
Male	170 (57.0)	0 (0)	170 (57.2)
Female	128 (43.0)	1 (100)	127 (42.6)
**Residence**			
Ebeye	261 (87.6)	1 (100)	260 (87.5)
Carlos	4 (1.3)	0 (0)	4 (1.3)
Ebadon	4 (1.3)	0 (0)	4 (1.3)
Mejatto	8 (2.7)	0 (0)	8 (2.7)
Santo	21 (7.0)	0 (0)	21 (7.0)
**Place of birth**			
Hospital^a^	270 (90.6)	1 (100)	269 (90.6)
Primary care facility^b^	26 (8.7)	0 (0)	26 (8.8)
Home	2 (0.7)	0 (0)	2 (0.7)
**HBV vaccination**			
Timely birth dose < 24 hours	269 (90.3)	1 (100)	268 (90.2)
Birth dose given at > 24 hours	29 (9.7)	0 (0)	29 (9.8)
Third dose at < 6 months	173 (58.1)	1 (100)	172 (57.9)
Third dose at > 6 months	125 (41.9)	0 (0)	125 (42.1)

HBV: hepatitis B virus.

Analysis of statistical significance was not done due to insufficient numbers of seropositive children.

^a^ Includes those born in hospitals outside Kwajalein Atoll such as Majuro Hospital.

^b^ Includes all dispensaries outside Ebeye.

As two of the mothers had twin deliveries, a total of 296 biological mothers were included in the seroprevalence analysis; the majority (97.6%) were aged 25 years or over. The prevalence of chronic HBV infection among mothers during pregnancy was 6.8% (95% CI: 3.90–9.62%). Around one fifth (*n* = 62; 20.9%) had completed a three-dose course of HBV vaccination (**Table 2**). All 20 mothers with chronic HBV infection were aged 25 years or over, and 90.0% (18/20) had not been vaccinated against HBV, not even partially. Among this group of mothers, only education level was associated with chronic HBV infection (**Table 2**). The outer island of Santo had the highest proportion of missing antenatal records (16/21, 76.2%).

**Table 2:** Characteristics of the biological mothers of first grade children from Kwajalein Atoll included in the 2016–2017 national seroprevalence study of HBV infection, by infection status

Characteristic	All mothers (*n* = 296) *n*(%)	Seropositive (*n* = 20) *n*(%)	Seronegative (*n* = 276) *n*(%)	*P*
**Age (years)**				
< 25	7 (2.4)	0 (0)	7 (2.5)	1.00
≥ 25	289 (97.6)	20 (100)	269 (97.5)	-
**Education**				
Primary	28 (9.5)	3 (15.0)	25 (9.1)	0.03
Some high school	140 (47.3)	4 (20.0)	136 (49.3)	-
Completed high school/college	128 (43.2)	13 (65.0)	115 (41.7)	-
**Employment**				
Employed	79 (26.7)	7 (35.0)	72 (26.1)	0.43
Not employed	217 (73.3)	13 (65.0)	204 (73.1)	-
**HBV vaccination status**				
Vaccinated	62 (20.9)	2 (10.0)	60 (21.7)	0.27
Not vaccinated	234 (79.1)	18 (90.0)	216 (78.3)	-
**No. of return antenatal visits**				
< 3	27 (9.1)	0 (0)	27 (9.8)	0.16
≥ 3	209 (70.6)	18 (90.0)	191 (69.2)	-
Unknown^a^	60 (20.3)	2 (10.0)	58 (21.0)	-

HBV: hepatitis B virus.

*P*-values compare the proportions of mothers who are seropositive and seronegative using Fisher’s exact test.

^a^ Antenatal records were missing for the pregnancy of interest and HBV status was assessed from a subsequent pregnancy.

Timely completion of HBV vaccination at birth and at 6 months of age were both significantly associated with place of residence (*P* < 0.001), place of birth (*P* < 0.001) and number of return antenatal visits (*P* < 0.001). The proportion of children receiving a timely birth dose of HBV vaccine was much lower in Santo (3/21; 14.3%) compared with the other areas (> 95.0%). The proportion of children who had their third dose of HBV vaccine by 6 months of age was greatest in Ebeye (166/259 births; 64.1%); in the other regions this proportion dropped to 25.0% or below. Completion of the third dose by 6 months of age was also significantly associated with maternal employment (**Table 3**).

**Table 3:** Sociodemographic factors associated with timely birth dose of HBV vaccine and completion of three-dose vaccination schedule by 6 months of age in schoolchildren aged 6–8 years in Kwajalein Atoll

Characteristic	Total^a^ *n* = 296	Timely birth dose given (*n* = 296)^a^ *n*(%)	Birth dose given at > 24 hours (*n* = 29) *n*(%)	*P*	Timely third dose (*n* = 173) *n*(%)	Third dose given at > 6 months (*n* = 123) *n*(%)	*P*
**Child’s age (years)**							
6	175 (59.1)	156 (58.4)	19 (65.5)	0.774	108 (62.4)	67 (54.5)	0.070
7	101 (34.1)	92 (34.5)	9 (31.0)		58 (33.5)	43 (35.0)	
8	20 (6.8)	19 (7.1)	1 (3.5)		7 (4.1)	13 (10.6)	
**Residence**							
Ebeye	259 (87.5)	248 (92.9)	11 (37.9)	< 0.001	166 (96.0)	93 (75.6)	< 0.001
Carlos	4 (1.4)	4 (1.5)	0		1 (0.6)	3 (2.4)	
Ebadon	4 (1.4)	4 (1.5)	0		1 (0.6)	3 (2.4)	
Mejatto	8 (2.7)	8 (3.0)	0	-	2 (1.2)	6 (4.9)	-
Santo	21 (7.1)	3 (1.1)	18 (62.1)	-	3 (1.7)	18 (14.6)	-
**Place of birth**							
Hospital^b^	268 (90.5)	264 (98.9)	4 (13.8)	< 0.001	170 (98.3)	98 (79.7)	< 0.001
Primary care facility^c^	26 (8.8)	1 (0.4)	25 (86.2)		2 (1.2)	24 (19.5)	
Home	2 (0.7)	2 (0.8)	0		1 (0.6)	1 (0.8)	
**Maternal age (years)**							
< 25	7 (2.5)	7 (2.6)	0	1.00	5 (2.9)	2 (1.6)	0.703
≥ 25	289 (97.6)	260 (97.4)	29 (100)		168 (97.1)	121 (98.4)	
**Education**							
Primary	28 (9.5)	27 (10.1)	1 (3.5)	0.381	16 (9.3)	12 (9.8)	0.741
Some high school	140 (47.3)	123 (46.1)	17 (58.6)	-	79 (45.1)	61 (49.6)	
Completed high school/college	128 (43.2)	117 (43.8)	11 (37.9)		78 (45.7)	50 (40.7)	
**Employment**							
Employed	79 (26.7)	69 (25.8)	10 (34.5)	0.376	54 (31.2)	25 (20.3)	0.045
Not employed	217 (73.3)	198 (74.2)	19 (65.5)		119 (68.8)	98 (79.7)	
**Maternal HBV vaccination status**							
Vaccinated	62 (21.0)	58 (21.7)	4 (13.8)	0.471	36 (20.8)	26 (21.1)	1.00
Not vaccinated	234 (79.1)	209 (78.3)	25 (86.2)		137 (79.2)	97 (78.9)	
**No. of return antenatal visits**							
< 3	27 (9.1)	24 (9.0)	3 (10.3)	< 0.001	11 (6.4)	16 (13.0)	< 0.001
≥ 3	209 (70.6)	205 (76.8)	4 (13.8)		138 (79.8)	71 (57.7)	
Unknown	60 (20.3)	38 (14.2)	22 (75.9)		24 (13.9)	36 (29.3)	
**Maternal HBV status**							
Negative	276 (93.2)	248 (92.9)	28 (96.6)	0.705	159 (91.9)	117 (95.1)	0.351
Positive	20 (6.8)	19 (7.1)	1 (3.5)		14 (8.1)	6 (4.9)	
**Maternal knowledge (out of 10)**							
Good, ≥ 6	153 (51.7)	137 (51.3)	16 (55.2)	0.845	91 (52.6)	62 (50.4)	0.725
Poor, < 6	143 (48.3)	130 (48.7)	13 (44.8)		82 (47.4)	61 (49.6)	
**Maternal attitude (out of 6)**							
Positive, ≥ 4	286 (96.6)	257 (96.3)	29 (100)	0.606	165 (95.4)	121 (98.4)	0.203
Negative, < 4	10 (3.4)	10 (3.8)	0		8 (4.6)	2 (1.6)	

HBV: hepatitis B virus.

^a^ For the purposes of analysing the participants as mother–child pairs, the two sets of twin children in this study were assumed to have the same vaccination status. The total reflects the number of mother–child pairs, rather than the number of children.

^b^ Includes those born in hospitals outside Kwajalein Atoll such as Majuro Hospital and hospitals in other countries.

^c^ Includes all dispensaries outside Ebeye.

### Knowledge and attitudes

The 360 mothers who were interviewed had a mean age of 35 years (range, 20–51 years; SD = 7). The majority (89.2%) were from Ebeye. Although most mothers (84.2%) were aware of HBV, knowledge about modes of transmission, vaccination and treatment was generally much lower (**Table 4**). Although around half of mothers (53.1%) scored ≥ 6 in the knowledge survey, the mean knowledge score was low (mean, 5.5; SD = 3.3). Questions relating to awareness of the potential complications of HBV infection and the availability of treatment received the fewest “yes” answers (**Table 4**). In contrast, responses to the six attitude questions were almost all positive, with 96.9% of mothers scoring ≥ 4; the mean attitude score was 5.9 (SD = 0.94). Questions relating to vaccination of children and antenatal screening received the highest proportion of positive responses (**Table 4**).

**Table 4:** Knowledge and attitudes towards HBV infection and vaccination among mothers on Kwajalein Atoll, 2016–2017 (*n* = 360)

Question topic	Proportion of “yes” answers % (95% CI)
**Knowledge items:**	
HBV infection	84.2 (80.0–87.8)
Complications such as liver cancer	44.2 (39.0–49.5)
Transmission through blood transfusion	50.3 (45.0–55.6)
Transmission through unprotected sexual intercourse	50.3 (45.0–55.6)
Mother-to-child transmission	60.8 (55.6–65.9)
Prevention of transmission through timely HBV vaccine birth dose	51.7 (46.4–56.9)
Asymptomatic nature of HBV infection	53.1 (47.8–58.3)
Ability to cause jaundice	73.3 (68.4–77.8)
Long-term complications for children infected perinatally	46.4 (41.1–51.7)
Availability of treatment for HBV infection	38.3 (33.3–43.6)
**Total with:**	
Good knowledge (knowledge score ≥ 6)	53.1 (47.8–58.3)
Poor knowledge (knowledge score < 6)	46.9 (41.7–52.2)
**Mean score**	5.5 (SD = 3.3)
**Attitude items (positive attitude):**	
Vaccination	95.8 (93.2–97.6)
Recommending vaccination to others	96.1 (93.6–97.9)
Being screened during pregnancy (antenatal visit)	96.7 (94.2–98.2)
Allowing child to be vaccinated	97.5 (95.3–98.9)
Allowing child to receive immunoglobulin treatment	96.3 (93.9–98.1)
Allowing child to be screened for HBV infection postnatally (first 12 months)	96.7 (94.2–98.3)
**Total with:**	
Positive attitude (attitude score ≥ 4)	96.9 (94.6–98.5)
Negative attitude (attitude score < 4)	0.03 (0.02–0.05)
**Mean score**	5.9 (SD = 0.94)

CI: confidence interval; HBV: hepatitis B virus; SD: standard deviation.

## Discussion

This study found the seroprevalence of HBV infection among first grade children on Kwajalein Atoll to be very low (0.3%) and confirms that good progress towards the 2030 target of < 0.1% is being made. (*5*) While the prevalence of chronic HBV infection among the children’s mothers was higher, at 6.8%, this figure is lower than that reported in a previous study conducted in perinatal women, (*17*) emphasizing the importance of subnational data for fully understanding the epidemiology of the HBV burden in the Marshall Islands. Our results also suggest that timely birth dose vaccination on Kwajalein Atoll may have reduced mother-to-child transmission of HBV despite the absence of hepatitis B immunoglobulin treatment and lower-than-optimal HBV vaccination coverage levels (i.e. below the 95% target for the Western Pacific Region). (*17*)

Timely HBV birth dose vaccine coverage for the first grade children included in this study was 90%; the three-dose coverage at 6 months was 58%. Both timely birth dose and three-dose completion at 6 months were significantly associated with factors related to place of birth and residence, with children living in areas outside the main urban centre of Ebeye more likely to miss out on their HBV vaccinations. This suggests that increasing the coverage of timely birth doses in these more remote areas, where there was less than 100% coverage and ensuring completion of three doses before 6 months of age in all areas will promote further reduction in childhood infection rates.

Interventions that have the potential to increase HBV vaccine coverage in remote settings such as Kwajalein Atoll include provision of out-of-cold-chain vaccines. Studies have shown that HBV vaccines are heat stable, (*24*) and that out-of-cold-chain vaccines can improve uptake in low-resource settings where refrigeration may be limited. (*25*, *26*) This strategy has been shown to be a potentially cost-effective approach for PICs and should be considered for the Marshall Islands as a whole. (*27*) Introduction of HBV immunoglobulin use in neonates may also further reduce mother-to-child transmission and should also be considered for the Marshall Islands.

In this study, all the mothers who had chronic HBV infection were born before the introduction of routine childhood immunization on Kwajalein Atoll. Based on the assumption that rates of maternal HBV infection are a useful proxy for disease prevalence in the adult population, the relatively high prevalence of HBV infection in mothers observed in this study suggests that ongoing transmission on the atoll is likely, possibly through unprotected sexual contact. Treatment for chronic HBV infections is not widely available in the Marshall Islands, (*13*) and, as demonstrated by this study, knowledge levels surrounding both the disease and its treatment among adults are low. Without greater awareness and treatment, a large number of adults in the Marshall Islands remain at risk of complications associated with chronic HBV infection, such as hepatocellular carcinoma. (*19*, *28*)

The findings of poor knowledge of HBV infection among women on the atoll indicate an important need for culturally appropriate public education and awareness-raising interventions to improve vaccination rates. Antenatal visits appear to be important settings for educating women. Prenatal HBV screening programmes provide a valuable opportunity to identify chronic HBV infections and to immunize unvaccinated pregnant women. Strategies for expanding screening of partners during and following pregnancy to encourage vaccine uptake should be considered by the national immunization programme. Integrated strategies for increasing access to prenatal care and screening for co-infections should also be explored. Standardized reporting of vaccination coverage and seroprevalence survey data is needed to ensure that subnational data are available for monitoring purposes.

In common with the situation reported in other high-burden settings, this study found that despite relatively poor disease knowledge, attitudes towards vaccination of infants were predominantly positive. (*29*) This finding, coupled with the observation that high levels of knowledge are not always positively correlated with favourable attitudes towards vaccination, (*30*) suggests that the barriers to screening and vaccination in this setting are complex and require further investigation.

While our study findings on the prevalence of HBV infection are not generalizable outside this setting, when viewed in the context of the data on vaccination coverage, they do provide some information that may be applicable to other PICs. Moreover, similarities between our knowledge and attitudes survey results and those derived from work conducted in other Pacific settings also indicate some commonality. (*19*, *21*) Our finding that seropositivity was more common in mothers with higher measures of socioeconomic status (higher education) requires further investigation.

This study has several limitations. The seroprevalence survey results for the children in this study date from 2016–2017 and for their mothers from 6–8 years before this when they were pregnant with these children. As such, the seroprevalence data may not represent the current HBV disease status on Kwajalein Atoll. As new interventions for HBV disease control have since been implemented, the estimates presented here are likely to be higher than present-day levels. Use of school records to identify study participants excludes children who are not enrolled in school, which may limit the generalizability of the results. However, this is not likely to be significant as enrolment rates on the atoll are higher than national estimates. For mothers with no antenatal record for the pregnancy of interest, the serostatus during subsequent pregnancies was used. This may have resulted in some misclassification as the serostatus may have changed between pregnancies. In addition, antenatal records for women from the outer islands were more likely to be missing than those for mothers from the main atoll, which may have introduced confounding by place of residence. Interviews for the knowledge and attitudes survey were conducted by public health nurses, which may have created some social desirability bias in the responses, particularly those relating to attitudes.

### Conclusion

This study showed significant progress towards regional targets for hepatitis B control on Kwajalein Atoll of the Marshall Islands and a reduction in the mother-to-child transmission of HBV through the timely administration of HBV vaccine birth dose. To ensure ongoing timely completion of HBV vaccination schedules, greater vaccine accessibility is required, and it is recommended that consideration be given to the use of out-of-cold-chain HBV vaccines in the national immunization programme. Reduction of disease prevalence among adults will require culturally appropriate public education activities and innovative approaches focused on women with poor vaccine uptake and low levels of knowledge. Prenatal visits provide a critical opportunity for screening, vaccination and education. Policies and integrated approaches for improving prenatal vaccination coverage and expanded screening should be considered to improve vaccination uptake among adults on Kwajalein Atoll and the Marshall Islands. Further research is needed to explore barriers to vaccination in adults.
